# Abdominal Aortic Aneurysm Formation with a Focus on Vascular Smooth Muscle Cells

**DOI:** 10.3390/life12020191

**Published:** 2022-01-27

**Authors:** Guoqing Qian, Oluwaseun Adeyanju, Ayobami Olajuyin, Xia Guo

**Affiliations:** Department of Cellular and Molecular Biology, The University of Texas Health Science Center at Tyler, Tyler, TX 75708, USA; guoqing.qian@uthct.edu (G.Q.); oluwaseun.adeyanju@uthct.edu (O.A.); ayobami.olajuyin@uthct.edu (A.O.)

**Keywords:** abdominal aortic aneurysm, inflammation, vascular smooth muscle cell, oxidative stress, apoptosis, extracellular matrix, phenotypic change

## Abstract

Abdominal aortic aneurysm (AAA) is a lethal degenerative vascular disease that affects, mostly, the elder population, with a high mortality rate (>80%) upon rupture. It features a dilation of the aortic diameter to larger than 30 mm or more than 50%. Diverse pathological processes are involved in the development of AAA, including aortic wall inflammation, elastin breakdown, oxidative stress, smooth muscle cell (SMC) phenotypic switching and dysfunction, and extracellular matrix degradation. With open surgery being the only therapeutic option up to date, the lack of pharmaceutical treatment approach calls for identifying novel and effective targets and further understanding the pathological process of AAA. Both lifestyle and genetic predisposition have an important role in increasing the risk of AAA. Several cell types are closely related to the pathogenesis of AAA. Among them, vascular SMCs (VSMCs) are gaining much attention as a critical contributor for AAA initiation and/or progression. In this review, we summarize what is known about AAA, including the risk factors, the pathophysiology, and the established animal models of AAA. In particular, we focus on the VSMC phenotypic switching and dysfunction in AAA formation. Further understanding the regulation of VSMC phenotypic changes may provide novel therapeutic targets for the treatment or prevention of AAA.

## 1. Introduction

**Figure 1:**
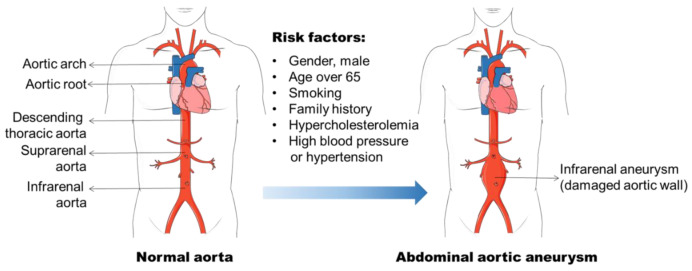
Abdominal aortic aneurysm formation and its risk factors. Abdominal aortic aneurysm (AAA) occurs in the infra-renal segment with a diameter exceeding 3.0 cm. The risk factors, including male gender, aging, smoking, and hypercholesterolemia, etc., have been found to be related to AAA initiation and progression.

Aneurysm is the term for a dilated blood vessel with a diameter at least 1.5 times its normal size [1]. According to the locations, aneurysms can be divided into three major types, i.e., cerebral aneurysm (brain), aortic aneurysm (AA, aorta), and popliteal aneurysm (popliteal artery) [2,3,4]. The aorta is the largest blood vessel in the body and the AA is categorized into two main types: thoracic aortic aneurysm (TAA, in the chest) and abdominal aortic aneurysm (AAA, in the abdomen) [3]. AAA usually occurs in the infra-renal segment with a diameter exceeding 3.0 cm (Figure 1) [3]. AAA is the most common aneurysm and predominantly affects men aged 65 years and older [5,6].

AAA diagnosis remains a challenge because it does not present with any symptoms, nor can it be detected by a mere physical examination [7]. With the growing of the aortic diameter, the risk of AAA rupture increases [8]. The rupture of AAA results in profound internal bleeding with a mortality around 80% [9]. The ultrasound screening of the high-risk populations (men of 65-years and older) has been demonstrated to be an effective approach to prevent the AAA related mortality [10,11]. However, it is costly and not appropriate for the assessment of AAA progression. The treatment option for patients with large (≥55 mm), rapidly growing (>10 mm), or symptomatic AAAs remains endovascular exclusion or open surgery [5], although the postsurgical mortality for emergency operations stays at around 50% [12]. Patients who have small AAAs (<55 mm) are not beneficial from surgical repair [7]. Currently, there is no specific drug available to prevent or reverse AAA progression.

**Figure 2:**
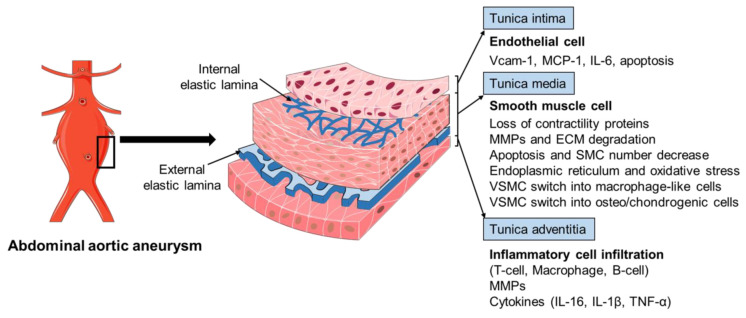
The pathophysiology of abdominal aortic aneurysm. The pathophysiology of abdominal aortic aneurysm (AAA) is a complicated process, involving the endothelial cell (EC) dysfunction with increased expression of adhesion molecules and chemokines, vascular smooth muscle cell (VSMC) phenotypic changes and dysfunction, inflammatory cell infiltration into the aortic wall, oxidative stress, and extracellular matrix (ECM) remodeling. Various mediators are involved in this process, including vascular cell adhesion molecule 1 (Vcam-1), monocyte chemoattractant protein 1 (MCP-1), interleukin-6 (IL-6), interleukin-1β (IL-1β), matrix metalloproteinases (MMPs), and tumor necrosis factor-α (TNF-α).

The pathophysiology of AAA is complex, involving the increased expression of endothelial cell (EC) adhesion molecules and chemokines, the inflammatory cell infiltration into the aortic wall, vascular smooth muscle cell (VSMC) dysfunction, aortic extracellular matrix (ECM) remodeling, oxidative stress, and the formation of intraluminal thrombus (Figure 2) [3,6,13]. The mechanisms of the AAA initiation and progression remain incompletely understood. The current review discusses the formation and progression of AAA with a focus on VSMC phenotypic switching and dysfunction.

## 2. AAA Formation

### 2.1. Risk Factors for AAA

The identification of risk factors for AAA formation provides strategies for AAA prevention and therapy. In the past few decades, various factors including male gender, aging, cigarette smoking, and elevated blood cholesterol level, etc., have been found to be related to AAA initiation and progression (Figure 1) [6,14,15]. The incidence of AAA is directly proportional to an increase in age, and AAA is a prominent cause of death in older people from 65 years of age [5]. It is often seen in older men, and the ratio of male and female patients ranges 3.5–6:1 based on various screening studies [16,17]. Therefore, the men with an age of more than 65 years old are the high-risk population for AAA development. 

Smoking has been recognized as a strong risk factor for AAA in various epidemiological studies [14,16,18]. Approximately 87% of the men with AAA were reported to be current or ex-smokers in a screening study of AAA among 65-year-old Swedish men with an odd ratio of 3.5 (95%CI: 2.4–5.1) [19]. The nicotine in plasma contributes to AAA progression, which has been confirmed in Apolipoprotein E deficient (ApoE−/−) mouse model and elastase-perfusion model of AAA [20,21,22]. The mechanisms underlying smoking induced AAA involve VSMC dysfunction and inflammatory cell function that have been discussed in Norman, P. E. and Curci, J. A. published review [20]. Hypercholesterolemia is a common facilitating cause of AAA, although there is limited knowledge as to its role in the development of AAA [15]. The abdominal aortic region is prone to the atherosclerotic lesion, which is found in virtually all cases of human AAA patients and that atherosclerosis is involved in the process of aortic dilation. Additional evidence to support the important role of hypercholesterolemia in aneurysm formation is the increased AAA formation in angiotensin II (Ang II) infused ApoE−/− mice (hypercholesterolemia mouse model) compared with wild-type (WT) C57BL/6 mice [23]. Genetic analyses of AAA from population screening and gene-associated studies show that different genetic risk factors are involved in the pathogenesis of AAA [24]. A heritability of 70% was reported in a large twin study by Jesper Swedenborg’s research group [25]. Several other studies have found that the risk of AAA in first-degree relatives of affected individuals was almost doubled [26,27,28]. Two loci for AAA have been identified and mapped on chromosome 19q13 and 4q31 [29,30]. The genome wide association studies (GWAS) have identified associations of single nucleotide polymorphisms (SNPs) with AAAs, such as CDKN2BAS1 rs10757274, the low-density lipoprotein receptor (LDLR) rs6511720, and DAB2IP rs10985349 [31,32]. In addition, other factors including DNA methylation, hypertension, and viral infections (e.g., cytomegalovirus, CMV) are also associated with AAA pathogenesis [33,34,35,36,37].

### 2.2. Histopathology of AAA

The histopathology of AAA is characterized by aortic wall inflammation, EC alteration, SMC dysfunction, oxidative stress, and ECM degradation, which cause a progressive luminal dilation, and finally a rupture (Figure 2) [6,13,38,39]. The aortic wall inflammation with the infiltration of inflammatory cells, including T-cells, B-cells, and macrophages, are essential features of AAA (Figure 2) [40,41,42]. In clinical and experimental AAA, a number of pro-inflammatory cytokines are increased, such as monocyte chemoattractant protein 1 (MCP-1), interleukin-1β (IL-1β), IL-6, and tumor necrosis factor-α (TNF-α). Polymorphisms in inflammatory cytokines may affect the production of these cytokines and, therefore, influence the pathogenesis of AAA [23,43,44]. SNPs (rs1800795 and rs1800796) in the IL-6 promoter have been linked with the development of AAA [44,45]. These inflammatory mediators play a critical role in inflammatory cell infiltration, which promotes an inflammatory response and subsequent SMC dysfunction, ECM degradation (mainly through matrix metalloproteinases, MMPs), and eventual AAA formation [42,46,47,48]. In addition, fragments of the aortic wall degradation serve as attracting agents for macrophage infiltration into the aortic wall to initiate the immune responses and AAA formation [49]. Dysregulation of EC function is another important factor implicated in AAA initiation and/or progression [50,51]. The increased EC expression of MCP-1 and vascular cell adhesion molecule 1 (Vcam-1) recruits macrophages into the aortic wall and leads to ECM degradation and, finally, aneurysm formation (Figure 2). In addition, EC apoptosis with the reduced expression of endothelial nitric oxide synthase (eNOS) also facilitates AAA formation by affecting the activity of NO, which is important in the stability of vascular tone, blood pressure, and SMC relaxation. 

VSMCs in the tunica media represent the most abundant cells in aorta arteries. The important role and phenotypic modulation of VSMCs in AAA have been explored extensively, including the SMC genetic modulation, SMC-mediated ECM production and degradation, SMC-upregulated inflammatory response, and SMC-modulated oxidative stress and apoptosis (Figure 2) [3,13,52,53,54,55]. The cellular and molecular mechanisms of SMCs mediated AAA development will be discussed in more detail in the following section. 

## 3. Vascular Smooth Muscle Cells in AAA Formation

### 3.1. VSMC Phenotypic Plasticity

The artery wall is made up of three layers, including the tunica intima (ECs), tunica media (mainly VSMCs), and tunica adventitia (fibroblasts and extracellular matrix) (Figure 2) [56]. During vascular development, precursor VSMCs are recruited to the endothelial vascular network and further differentiate into mature SMCs through various signaling pathways, such as transforming growth factor-β (TGF-β)/Smads, platelet-derived growth factor-BB (PDGF-BB), Wnt/Notch, histone deacetylases (HDACs)/epigenetics, micro-RNAs, etc. [57,58,59]. In a healthy vessel wall, VSMCs play a major role in maintaining vascular tone to mediate blood pressure and blood flow through their contraction and dilatation capability [56,60]. Accumulating evidence shows that SMCs’ genetic and epigenetic modulation in vasculogenesis during embryo development is a major mechanism in AAA formation. The embryological origins of SMCs in the aortic arch and descending aorta are different [58,60,61]. Compared with the aortic arch SMCs derived from the neural crest, those descending aortic SMCs instead are derived from the mesoderm with less elastic lamellae formed during vascular development. There are also differences in the cellular content and genetic activity, which renders the infra-renal region an area prone to aneurysm. For example, the mesoderm derived SMCs are more responsive to the cytokines IL-1β, which can upregulate MMPs’ expression and further ECM degradation. In addition, TGF-β induction increased DNA synthesis and collagen production in neural crest-derived SMCs but not in mesoderm-derived SMCs. 

SMCs are highly plastic and undergo significant changes between two phenotypes, i.e., a rather ‘dormant’ one with differentiated SMCs, and a proliferating/synthetic one with dedifferentiated SMCs in response to stress signals [56,60,62]. Differentiated SMCs are spindle shaped and express high levels of contractile proteins, such as α-smooth muscle actin (α-SMA), SM myosin heavy chain (SMMHC), smooth muscle 22α (SM22α), SM-calponin (CNN), and smoothelin-B [59]. Under pathological conditions, SMCs could be induced by TGF-β, PDGF-BB, Ang II, etc., and change into a dedifferentiated phenotype with low levels of contractile proteins but high levels of molecules associated with proliferation, migration, fibrosis, and inflammation [60,63,64,65]. Mechanical injury, atherosclerosis, hypertension, as well as aneurysm are all related with the SMC phenotypic changes [56,64,65,66,67]. 

### 3.2. VSMC Phenotypic Modulation in AAA

#### 3.2.1. VSMC Contractility and TGF-β

VSMCs play a central role in aneurysm formation. VSMCs in the healthy vessel wall display a contractile phenotype to maintain the vascular tone. The loss of SMC contractile function may alter the vascular tone and increase the aortic wall stress to promote aneurysm formation [52,64,68]. The mutations in genes encoding SMC contractile proteins have been reported to be associated with AAA development. The TGF-β pathway components, including the receptors and Smad proteins, are involved in SMC contractility through upregulating the expression of SMC contractile proteins (α-SMA, SMMHC, and CNN, etc.) [68,69,70,71]. TGF-β neutralization has been shown to augment Ang II induced aneurysms in both thoracic and abdominal regions [72,73]. Consistently, the disruption of the TGF-β receptors in SMCs impairs their contractile ability and results in aneurysm [70]. The genetic deletion of Smad3 causes extensive aneurysm formation with elastic fiber fragmentation, collagen fiber reorganization, and vessel inflammation in the calcium chloride (CaCl_2_)-induced mouse AAA model [71]. In addition, a previous study showed a remarkable downregulation of TGF-β receptor 2 (TGFBR2) in human AAA biopsies. Eleven of twelve AAA biopsies demonstrated TGFBR2 exon 8 deletion with a marked downregulation of TGFBR2 in AAA biopsies [74]. Further, an AAA case-control study from a Dutch population also reported that SNPs in both TGFBR1 (rs1626340) and TGFBR2 (rs1036095 and rs4522809) are associated with AAA prevalence [32,75]. These studies demonstrate that TGF-β signaling is necessary to sustain the structural integrity of SMCs and prevent aortic dilatation during AAA. A recent lineage tracing study using SMC-specific deletion of TGFBR2 provides consistent and convincing in vivo evidence. Those mice lacking TGF-β signaling in their SMCs on an ApoE−/− background developed AAAs after 4 months’ feeding with a high cholesterol high fat diet, while the wild-type counterparts did not. The trans-differentiation of a subset of contractile SMCs into mesenchymal stem cell-like cells might account for such phenomenon since the latter can further give rise to other cell types, including osteoblasts/chondrocytes, adipocytes, and macrophages [76]. 

Another layer of control in VSMC phenotypic changes lies in micro-RNA (miRNA). micro-RNAs are small and non-coding RNAs, which function to repress gene expression by degrading messenger RNAs or mimicking small interfering RNAs (siRNAs) to inhibit translation. The miR-143/145 cluster highly expressed in VSMCs has been shown to be most abundantly expressed in the heart and the aorta [77]. Serum response factor (SRF), myocardin, and Nkx2.5 are reported to induce the miR-143/145 expression in SMCs [78]. Overexpression of miR-145 upregulates the levels of SMC contractile proteins, including α-SMA, SMMHC, and CNN. Such a function of miR-145 in increasing SMCs’ contractility primarily acts through suppressing Kruppel-like transcription factor 4 (KLF4) and KLF5, which represses myocardin to downregulate VSMC differentiation marker genes [78]. In addition, the deregulation of miR-143/145 has also been implicated in human aneurysm development [79]. 

#### 3.2.2. SMCs Express Proteolytic Enzymes to Induce ECM Disorganization

Aortic aneurysm is a matrix degenerative disease with dilated blood vessels. Multiple studies have demonstrated that ECM degradation during AAA formation is actively mediated by VSMCs [6,13,52,64]. The ECM consists of elastin, collagen, fibronectin, and fibrillin. VSMCs produce elastin and collagen to resist vasodilation and rupture. On the other hand, VSMCs control the integrity and degradation of ECM by the release and maturation of MMPs and the tissue inhibitor of metalloproteinases (TIMPs) [54,56,80,81,82,83,84,85]. During the aneurysm formation, the breakdown of elastin results in SMC phenotypic changes characterized by an increased expression of MMPs, such as MMP-1, -2, -9, -13, and -14 [54,80]. These MMP proteins exert proteolytic activity towards elastin and collagen, which are essential in ECM degradation and disorganization during AAA formation. Increased expression/activity of MMP-2 and MMP-9 are of particular significance in degrading the extracellular matrix and weakening the aortic vascular wall in the context of AAA formation [81]. The gene polymorphic sites have been recognized in the promoter of a number of MMPs [82]. The substitution of cytosine with thymidine in the promoter region of MMP-9 increases its promoter activity [86], leading to upregulated MMP-9 in AAA patients. A similar substitution occurs in the polymorphic site in the promoter regions of MMPs -2, -3, -9, and -12 genes [87,88,89]. 

VSMCs modulate the MMP activities and ECM degradation through inhibiting the expression of TIMPs (TIMP-1, -2, and -3) [83,84,85]. The decreased TIMP expression or activity has been shown to mediate aneurysm development through resulting upregulated MMP activities and increased ECM degradation [90]. The TIMP1-deficient mice developed larger aneurysms compared to the WT mice after porcine pancreatic elastase (PPE) perfusion [91]. Additionally, the MMP/TIMP ratio is controlled by plasminogen activator inhibitors (PAIs) [92]. VSMCs also express a number of miRNAs to mediate ECM degradation and promote AAA [93,94,95]. The murine miR-712 or human/murine homolog miR205 have been shown to repress TIMP translation and, consequently, increase MMP activity and promote AAA development [95]. The upregulated expression of miRNAs, including miR-21, -133, and -378, have been found in aortic aneurysm tissues from mouse AAA models and human AAA patients [94]. HDACs, regulators of gene transcription, have been shown to modulate the expression of VSMC genes involved in AAA [96]. Consistently, the HDAC inhibitor MCT-1 decreases MMP2 expression and activity in VSMCs in vitro [97]. An increased expression of the HDAC profile has also been noted in aneurysm samples from AAA animal models and human patients [96]. Further, the HDAC inhibitors including MS-275, MC-1568, and MCT-1 have been found to improve ECM disorganization and inhibit aneurysm development in mouse AAA models [93,98]. In a study exploring the effects of calorie restriction on AAA development, Sirtuin 1 (SIRT1) expression in VSMCs was found critical for mediating the protective effects of calorie restriction against aortic aneurysm formation. The VSMC-specification knockout of SIRT1 abolished the protective effect of calorie restriction. Such an effect was attributed partly to the SIRT1–dependent deacetylation of histone H3 lysine 9 on the MMP2 promoter [99]. The results suggest SIRT1 as a novel regulator of VSMC ECM remodeling during energy restriction in the context of AAA development.

#### 3.2.3. Endoplasmic Reticulum Stress and Oxidative Stress

The increased endoplasmic reticulum (ER) stress has been implicated in aortic aneurysm formation [100,101,102]. The inhibition of ER stress successfully decreases aneurysm formation in Ang II-induced mouse AAA models [102]. In the absence of ER stress, the transcription factor unspliced X box protein 1(XBP1u) is expressed to maintain VSMC contractile phenotype. XBP1u deficiency induced AAA formation in vivo with VSMC dedifferentiation and the increase of proinflammatory and proteolytic VSMCs [103]. ER stress triggers a thoracic aortic aneurysm and dissection (TAAD) formation through the C/EBP homologous protein (CHOP), which controls ER stress-induced apoptosis. CHOP deletion, therefore, has been shown to prevent SMC apoptosis and TAAD development [104]. 

Oxidative stress, as defined by the excess production of reactive oxygen species (ROS), promotes AAA development through multiple mechanisms, such as VSMC apoptosis induction, MMP activation, and pro-inflammatory cytokine production [13,53,93,105,106,107,108,109]. Antioxidant treatment via vitamin E administration lowered oxidative stress and inhibited AAA formation and rupture [105]. The ROS has also been found increased in SMCs and human AAA tissues [53,109,110,111]. Generally, scavenging ROS reduces the formation of AAA in mice and safeguards against aortic aneurysm development [110]. Elevated ROS has been correlated with increased HAT activity and augmented histone acetylation in SMCs, which promotes inflammatory changes in SMCs [109]. A previous in vivo study also showed that ROS contributes to inflammatory SMCs via upregulating cyclophilin, a pro-inflammatory mediator that promotes ERK1/2 phosphorylation and MMP-2 activation [112]. NADPH (nicotinamide adenine dinucleotide phosphate) oxidase, NOX, is a major source of ROS in AAA development [111]. The enhanced expression of NOX1, NOX2, NOX4, and NOX5 have been observed in human SMCs [111]. The mechanical stress is one of the inducers to activate NADPH oxidase to enhance ROS production in VSMCs, in addition to Ang II, PDGF, oxidized low-density lipoprotein (ox-LDL), and certain cytokines such as TNF-α. NOX enzymes have been well studied in AAA pathology [108]. The iNOS deficiency and NADPH oxidase inhibition by apocynin decreases NO(x) levels and suppresses aneurysm formation [110]. The genetic removal of p47^*phox*^, a subunit of NADPH oxidase, reduces AAA incidence in the Ang II induced mouse AAA model [113]. NOX1-mediated ROS generation has been reported to lower contractile protein expression in aneurysms [114]. NOX4-mediated oxidative stress has been shown to be implicated in SMC apoptosis, which is a vital process in AAA formation [111]. Taken together, SMC oxidative stress is an important pathological event in the development of AAA; its effects diverge depending on the activated components and the context of aneurysmal induction.

#### 3.2.4. Apoptosis and SMC Loss

A fundamental difference between the healthy vessel wall and the aneurysmal wall lies in the decreased number of VSMCs [6,13,52]. The reduced VSMC number in the aortic wall attenuates their abilities in producing connective tissue and repairing elastin breaks, which induces AAA formation. SMC apoptosis is a major cause of SMC number reduction in AAA development [52,55,115]. The apoptotic SMCs have been frequently observed in the medial aortic wall of AAAs [52,115]. Apoptosis is associated with the generation of apoptotic bodies, which promotes calcium deposition if not cleared by phagocytosis. Calcium deposition in the aortic wall increases vessel wall stiffness and promotes AAA development [116]. SMC apoptosis could be triggered by inflammatory mediators, growth factors such as PDGF, cell stretch, hypoxia, and DNA damage [55,117]. In addition, SMC aging can ultimately progress into cell death [55,118]. The death mediator Fas/Fas ligand (FasL) signaling activates a caspase cascade (caspase-3 and -7) to induce the degradation of chromosomal DNA and apoptosis [119,120]. Further, the activation of Fas/FasL has been reported in SMCs of aneurysm tissues [121,122]. The serpin proteinase inhibitor B9 (serpinb9) has shown success in inhibiting VSMC cell apoptosis and ECM degradation induced by elastase [123]. In accord, the decreased expression of serpin proteinase inhibitors has been reported in AAA [124]. Growing evidence shows that ER stress and oxidative stress induce SMC apoptosis [104,111]. Autophagy related apoptosis is also implicated in the development of AAA. Recently, Lu et al. shows that the transcription factor EB (TFEB), a master regulator of autophagy, is critical for AAA development via regulating VSMC apoptosis. The VSMC-specific knockout of TFEB enhances VSMC apoptosis and promotes AAA formation in different preclinical models of AAA [125]. Consistently, the knockout of ATG7, a key regulator of autophagy, also aggravates angiotensin II-associated aortic remodeling [126]. 

A number of SNPs have been found through GWAS to be associated with SMC apoptosis in aneurysms, such as CDKN2BAS, DAB2IP, and LDL receptor-related protein 1 (LRP1). Knockdown or knockout of Cdkn2b, DAB2IP, and LRP1 in SMCs are capable of inducing SMC apoptosis and AAA formation [93]. 

Recent studies have demonstrated the critical roles of noncoding RNAs, including long noncoding RNA (lncRNA) and micro-RNA, in regulating VSMC apoptosis and aneurysm development [94,127,128]. miR-21 and miR-26a have been shown to protect against AAA formation through inhibiting VSMC apoptosis [22,94]. The overexpression of miR-21 through lentiviral transduction results in the decreased expression of phosphatase and tensin homolog (PTEN) and leads to the phosphorylation and activation of AKT, thus preventing VSMC apoptosis [22]. The inhibition of miR-26a via anti-miR transfection promoted H_2_O_2_-induced apoptosis of the human aortic SMCs [126]. In addition, lncRNA H19 has been found to be upregulated in Ang II and PPE induced AAA animal models, and knockdown of H19 inhibited the aneurysm formation in both AAA models. Mechanistically, H19 increases the expression of HIF1α, leading to increased VSMC apoptosis in aneurysm [129]. The overexpression of the lncRNA PVT1 has been found to induce VSMC apoptosis, elevate MMP-2 and MMP-9, and decrease TIMP-1 in Ang II-induced mouse AAA models. Conversely, blocking PVT1 reverses these effects in in vitro and in vivo settings [130]. Together, these findings emphasize apoptosis of SMCs as an important pathological event in the development of AAA. 

In addition to apoptosis, key mediators of necroptosis, including the receptor-interacting protein kinase 1 (RIPK1) and 3 (RIPK3), have been found to be increased in human AAA samples (especially in VSMCs) and in the elastase-induced mouse model of AAAs [131]. RIPK1/3 are inducers of aortic SMC necroptosis, silent mutation or inhibitors of RIPK1/3 could attenuate AAA expansion in elastase perfusion induced AAA models [131,132,133,134]. Increasing evidence also suggests a role of other cell death types such as ferroptosis and pyroptosis in the development of AAA [135]. In spite of limited knowledge, there is growing interest in targeting cell death pathways as a novel approach for AAA treatment.

#### 3.2.5. VSMCs Inflammatory Phenotypic Change and Transdifferentiation into Macrophage-like Cells

The aortic wall inflammation and inflammatory cell infiltration is an important component of AAA development [13,82,116,136,137]. Lineage tracing techniques have been employed to confirm that VSMCs can transdifferentiate into macrophage-like cells. The phenotypically transdifferentiated VSMCs comprised of approximately 30% of the macrophage population within the atherosclerosis lesions, as shown by lineage tracing in the ApoE−/− mice model [138]. The infiltrated macrophages are responsible for the upregulated cytokines in aneurysm tissues such as IL-1α, IL-1β, IL-6, and TNF-α, which could induce SMC phenotypic change. Interestingly, the inflammatory VSMCs also produce these cytokines, implying that the cytokine upregulation in aneurysms is likely also related to autocrine mechanism. The cytokine upregulation in the aortic wall could further induce MMP activation, VSMC apoptosis, and EC dysfunction to promote AAA formation [6,13]. The SET and MYND domain-containing 2 (SMYD2) methylation in aortic SMCs correlates with suppressed gene expression. The SMYD promoter region of SMCs is drastically hypomethylated in AAAs [139]. SMYD2 can methylate TNF-α and IL-6 promoters to suppress their transcription and inhibit NF-κB and ERK signaling pathways [140]. Therefore, hypomethylation of SMYD may result in increased inflammation and promote AAA development. In addition, miR-24 was shown to target chitinase 3-like 1 (Chi3l1, an inflammation marker of AAA disease progression) and decrease its expression. It suppresses inflammation by blocking IL-8 and MCP-1/CCL2 production by VSMCs. In accord, decreased plasma levels of miR-24 have been observed in AAA patients and murine AAA models [141]. 

#### 3.2.6. VSMC Phenotypic Switch into Osteo/Chondrogenic VSMCs

Vascular calcification, closely related with arterial stiffening, is characterized by the deposition of calcium phosphate crystals in the vessel wall [142,143,144]. It has been implicated in the development of a number of clinical diseases including chronic kidney disease, atherosclerosis, and hypertension. Recent studies show that vascular calcification is involved in AAA initiation and progression, and even rupture [144,145,146]. The detection of microcalcification assists the evaluation of the risk for AAA events [147,148]. Further, the active mineralization in AAA, as determined by the 18F-sodium fluoride uptake, has been shown to correlate with AAA progression [149,150]. The switching from contractile VSMCs to osteo/chondrogenic VSMCs is a key process in vessel wall calcification [151,152]. The osteo/chondrogenic VSMCs are featured by the increased expression of mineralization regulators, including osteocalcin, alkaline phosphatase, osteopontin, osteoprotegerin, etc., which are controlled by transcription factors such as Runt related transcription factor 2, Osterix, and SRY-box transcription factor 9 [151]. In bovine aortic SMCs, β-glycerophosphate (β-GP) stimulated calcium deposition and, meanwhile, decreased the expression of the elastic fibers [153]. These dysregulations of elastic fibers further cause calcification and decrease vascular elasticity, and finally affect the aneurysm development. The MMP-mediated elastin disorganization has also been shown to correlate with the calcium deposition on elastin fibers and the calcification during AAA [154]. The subsequent detachment of VSMCs from the elastic fibers, VSMC apoptosis, and the loss of matrix Gla protein (a calcification inhibitor), together contribute to the stimulation of the VSMC calcification event. 

## 4. Animal Models Used to Investigate SMC Phenotypic Change and AAA

To study the pathogenesis of AAA and test the potential of prospective therapies, a range of animal models have been developed in different species, such as mouse, rat, rabbit, and pig, etc. [155]. Rodent models, especially mouse models, of AAA have been widely used to explore the underlying mechanisms and therapies of aneurysm. The mouse model of Ang II infusion represents the most widely used animal model of AAA. Daugherty et al. firstly reported AAA formation in ApoE−/− mice when an osmotic mini pump with Ang II was implanted subcutaneously for 4 weeks [23]. The Ang II-induced mouse AAA model is established based on the hyperlipidemia mice, such as ApoE−/− or LDLR−/− male mice, that are susceptible to AAA formation with Ang II infusion, which yields an AAA incidence of approximately 80%, while in WT C57BL/6 mice the incidence is only 25%. The Ang II mouse model is technically easy and takes about one month for aneurysm formation. A common feature of the Ang II infusion model is the induction of arterial inflammation, with massive infiltration of leucocytes into the aorta, the degradation of the extracellular matrix, and smooth muscle phenotypic changes. Differing from the human AAA that occurs predominantly in the infrarenal aorta, the Ang II-induced aneurysms are present in suprarenal region and ascending aorta. However, the Ang II infusion model remains of great interest in the study of pharmaceutical intervention and genetic mutant effects in the development of AAA as it captures several important features of human AAA, including leukocyte infiltration, dissection, medial degeneration, and a close association with atherosclerosis [155].

Unlike the Ang II-induced mouse AAA model, the elastase-induced AAA model does not require hyperlipidemia. Thus, this model could be used to induce AAA in different species with results mimicking that seen in humans. In this model, a segment of surgically isolated aorta is exposed to PPE for 5 to 10 min, which causes cleaved aortic elastic fibers, and triggers an inflammatory response, SMC phenotypic changes, and, ultimately, aneurysm formation within two weeks. A simplified model has also been established by applying PPE at a higher concentration to the outside adventitia to cause acute inflammatory infiltration, ECM degradation, and aortic dilation [156,157]. The loss of VSMCs is directly proportional to the concentration of PPE used, further supporting the essential role of SMCs in AAA development. The PPE model is a frequently used AAA model because it is fast, genetic background independent, and feasible in various species. The weakness of this model includes failure of dissection and intraluminal thrombus induction in the aneurysm tissues [158]. In the CaCl_2_-induced AAA model, the aneurysm is achieved by applying cotton gauze soaked with CaCl_2_ to the peri-aortic wall for 15 to 20 min, followed by adding PBS on the aorta [116]. This model is easy to handle and feasible for different species without genetic background requirements. The pathology of this model involves calcification, inflammation, ECM degradation, and VSMC loss [159]. However, the dissection, rupture, and intraluminal thrombus do not develop in this model. A surgery-induced AAA model is also very useful, particularly in large animals, which could be utilized to test endovascular devices or surgical procedures. The frequently used approaches of the surgical model include an aortic patch, artificial aneurysm graft, intra-aortic PPE infusion, and aortic dissection with endovascular treatment [155]. The surgery-induced AAA model is technically challenging and more costly compared to small animal models, which limits the usage of this model. 

β-Aminopropionitrile, a lysyl oxidase (LOX) inhibitor, is capable of blocking LOX function in crosslinking elastin and collagen. The combination of β-Aminopropionitrile with the Ang II model or PPE model increases the incidence of AAA and the aneurysm diameter, even the rupture [160,161]. A high-fat diet combined with the Ang II model or PPE model has also been employed to enhance the aneurysm formation [161,162]. The PPE-induced aneurysm could be enhanced through a combined exposure to cigarette smoking, the most potent environmental risk factor for AAA. In addition, the combinations of the different AAA models have also been reported but will not be discussed in this review. It is evident that each AAA model has its own advantages and inherent limitations. Therefore, the selection of AAA animal model should take into account their respective features and the aim and objectives of the research. 

## 5. Application of Single-Cell RNA-Sequencing (scRNA Seq) in AAA Studies

scRNA seq emerges as a powerful approach to study transcriptome profile changes that are useful for identifying cellular clusters and exploring cellular responses in AAA. During AAA development, a myriad of cell types is involved, ranging from circulating immune cells to vascular resident cells (e.g., SMC). Recently, studies using scRNA seq have shed light on the heterogeneity and cellular responses of vascular cells in AAA progression. One recent study identified 17 clusters representing nine-cell lineages. Further Seurat clustering analysis identified four SMC subpopulations and five monocyte/macrophage subpopulations. During AAA progression, three major SMC subpopulations were proportionally decreased, whereas a small subpopulation was increased with downregulated SMC contractile markers and increased pro-inflammatory genes [163], suggesting phenotypic changes. Interestingly, scRNA seq analysis of lesioned aortas has identified macrophage-derived Netrin-1 as a robust inducer of the intracellular calcium flux and MMP3 activity by VSMCs, thereby it mediates the dynamic crosstalk between inflammation and ECM remodeling in AAA [164]. Single-cell analysis of the clinical aortic specimens from Marfan syndrome patients also revealed defective TGF-β signaling, i.e., downregulated TGFBR2 and Smad in a subset of SMCs [165]. Furthermore, an altered subpopulation of dedifferentiated proliferative SMCs was noted in the aortic tissues from Marfan syndrome patients but not from control subjects. These studies underscore the importance of the selective targeting of subgroups of VSMCs based on their transcriptome profiles. The scRNA seq analysis of AAA tissues are useful for dissecting the heterogeneity of cell subpopulations, deregulated signaling pathways, and cellular responses, as well as their interactions during AAA development. It also holds the key for identifying disease-relevant transcriptional signatures in VSMC-lineage cells, which might provide clues for disease predication, diagnosis, and prevention.

## 6. Conclusions

In summary, we present an overview of what is known about AAA, including the risk factors, the pathophysiology, and animal models used to explore the mechanism and therapies for AAA. AAA remains a serious threat to public health because of its high mortality after rupture. The endovascular exclusion and open surgical techniques are still the major treatment options for AAA. No drugs have been demonstrated to be effective in clinical trials. While mounting evidence from animal models and clinical research suggests inflammatory response and vascular remodeling as important pathological processes for AAA initiation, progression, and rupture, the VSMC phenotypic modulation is of particular interest as it is involved in both processes. Despite intensive research, it needs to be recognized that VSMC phenotypic switching is an evolving area, and the pathophysiology of VSMCs in AAA development remains incompletely understood. Further understanding the regulation of VSMC phenotypic changes and dysfunction in the development of AAA may help identify novel therapeutic targets for the treatment or prevention of AAA.

## Abbreviations

AAA	abdominal aortic aneurysm
Ang II	angiotensin II
α-SMA	α-smooth muscle actin
CNN	SM-calponin
EC	endothelial cell
ECM	extracellular matrix
HDACs	histone deacetylases
IL-1β	interleukin-1β
MCP-1	monocyte chemoattractant protein 1
MMP	matrix metalloproteinase
NOX	nicotinamide adenine dinucleotide phosphate oxidase
PDGF-BB	platelet-derived growth factor-BB
PPE	porcine pancreatic elastase
RIPK1/3	receptor-interacting protein kinase 1/3
ROS	reactive oxygen species
SMMHC	SM myosin heavy chain
SM22α	smooth muscle 22α
TAA	thoracic aortic aneurysm
TFEB	transcription factor EB
TGF-β	transforming growth factor-β
TGFBR1/2	TGF-β receptor 1/2
TIMPs	tissue inhibitor of metalloproteinases
TNF-α	tumor necrosis factor-α
Vcam-1	vascular cell adhesion molecule 1
VSMC	vascular smooth muscle cell
